# Response to Letter to the Editor

**DOI:** 10.1111/1759-7714.70122

**Published:** 2025-07-02

**Authors:** Alberto Busetto, Giorgio Cannone, Luigi Lione, Alessandro Bonis, Vincenzo Verzeletti, Michele Battistel, Alessandro Rebusso, Samuele Nicotra, Andrea Dell'Amore, Federico Rea

**Affiliations:** ^1^ Department of Cardiac, Thoracic, Vascular Sciences and Public Health University of Padua Padua Italy; ^2^ Thoracic Surgery Unit, Division of Surgery University – Hospital of Padua Padua Italy; ^3^ Department of Medicine University Radiology, University Hospital of Padova Padova Italy

Dear Editor,

1

It is with great pleasure that we read the letter addressing our recent article about the challenges and treatment strategies of chylothorax after thoracic surgery [1]. You correctly emphasize the role of MR lymphography as a valuable tool in the diagnostic algorithm of chylothorax [2]. It presents great advantages, such as the non‐invasiveness and the absence of administration of a contrast agent, reducing the risks for the patient. Moreover, it allows precise spatial resolution of the lymphatic system morphology and anomalies and the visualization of small collaterals associated with lymphatic leak localization [3].

While we agree with the usefulness of unenhanced MR lymphography in this context, in our article we proposed an overview of the most common strategies used for chylothorax treatment. MR lymphography is important in the diagnostic part of the algorithm, but it is not a treatment modality. In our surgical ward we have to be focused on rapid diagnostic assessment, and as Lipiodol lymphoangiography has also the potential to be a vessel sealant and thus to definitively treat the leakage, we prefer using this technique. Moreover, intraoperative management of relapsing and persistent leaks still requires a contrast agent for proper visualization of the leakage site, such as ICG [4]. As you already have stated, MR lymphography does not allow for the dynamic study of the lymphatic flow, and it becomes difficult to use it in the planning of the eventual future surgery [3, 4, 5].

Patients with chylothorax often undergo prolonged hospitalization, adversely affecting both clinical outcomes and the efficient allocation of healthcare resources. In this context, it is fair to state that MR machines are mainly used for elective diagnostics and it is difficult to obtain urgent MR diagnostics for hospitalized patients. Furthermore, MR is time consuming and has higher costs than lymphography. Most of all, this examination technique requires highly experienced and trained staff for interpretation of the findings, which are not promptly available in most institutions.

In conclusion, while we fully acknowledge the diagnostic value of unenhanced MR lymphography in the evaluation of chylothorax, its integration into routine clinical practice remains challenging in our setting due to organizational and staffing constraints. Ultimately, a balanced approach that considers both diagnostic precision and practical feasibility is essential. We appreciate the opportunity to engage in this important dialogue and welcome further discussion aimed at optimizing the management of this complex condition.

## Author Contributions

**Alberto Busetto:** conceptualization, data curation, methodology, investigation, writing – original draft. **Giorgio Cannone:** conceptualization, data curation, writing – review and editing. **Luigi Lione:** methodology, investigation. **Alessandro Bonis:** methodology, validation. **Vincenzo Verzeletti:** supervision, formal analysis, writing – review and editing. **Michele Battistel:** validation, visualization. **Alessandro Rebusso:** data curation, supervision, writing – review and editing. **Samuele Nicotra:** supervision, formal analysis, writing – review and editing. **Andrea Dell'Amore:** project administration, methodology, supervision, writing – review and editing. **Federico Rea:** supervision, formal analysis, project administration, writing – review and editing.

## Conflicts of Interest

The authors declare no conflicts of interest.

## Data Availability

The data that support the findings of this study are available on request from the corresponding author. The data are not publicly available due to privacy or ethical restrictions.
